# Long-Term Monitoring with Fiber Optics Distributed Temperature Sensing at Campi Flegrei: The Campi Flegrei Deep Drilling Project

**DOI:** 10.3390/s19051009

**Published:** 2019-02-27

**Authors:** Renato Somma, Claudia Troise, Luigi Zeni, Aldo Minardo, Alessandro Fedele, Maurizio Mirabile, Giuseppe De Natale

**Affiliations:** 1 Istituto Nazionale di Geofisica e Vulcanologia Sezione di Napoli, Via Diocleziano 328, 80124 Napoli, Italy; claudia.troise@ingv.it (C.T.); alessandro.fedele@ingv.it (A.F.); giuseppe.denatale@ingv.it (G.D.N.); 2 Università della Campania “Luigi Vanvitelli”- Dipartimento di Ingegneria, Via Roma 29, 81031 Aversa, Italy; luigi.zeni@unicampania.it (L.Z.); aldo.minardo@unicampania.it (A.M.); 3 Optosensing S.r.L., Via C. De Marco, Napoli 80134, Italy; mirabile@hpsystem.it

**Keywords:** fiber optics sensing, distributed temperature sensing, temperature calibration, volcanic monitoring, Campi Flegrei caldera

## Abstract

Monitoring volcanic phenomena is a key question, for both volcanological research and for civil protection purposes. This is particularly true in densely populated volcanic areas, like the Campi Flegrei caldera, which includes part of the large city of Naples (Italy). Borehole monitoring of volcanoes is the most promising way to improve classical methods of surface monitoring, although not commonly applied yet. Fiber optics technology is the most practical and suitable way to operate in such high temperature and aggressive environmental conditions. In this paper, we describe a fiber optics Distributed Temperature Sensing (DTS) sensor, which has been designed to continuously measure temperature all along a 500 m. deep well drilled in the west side of Naples (Bagnoli area), lying in the Campi Flegrei volcanic area. It has then been installed as part of the international ‘Campi Flegrei Deep Drilling Project’, and is continuously operating, giving insight on the time variation of temperature along the whole borehole depth. Such continuous monitoring of temperature can in turn indicate volcanic processes linked to magma dynamics and/or to changes in the hydrothermal system. The developed monitoring system, working at bottom temperatures higher than 100 °C, demonstrates the feasibility and effectiveness of using DTS for borehole volcanic monitoring.

## 1. Introduction

Monitoring hazardous geological processes is a fundamental tool for risk preparedness and mitigation efforts. Understanding and measuring the complex dynamics, still largely unknown, of geological hazard phenomena is then a fundamental tool, for both scientific and civil protection purposes. Opto-electronic sensors have gained much attention, since their early introduction, even in the field of geological hazard monitoring [1,2]. Distributed Optical Fiber Sensors (DOFSs) are among the most promising technologies for such monitoring purposes. A fundamental application of this opto-electronic technique, in such a research framework, is the continuous, distributed temperature monitoring along fiber optics cables. Multiple applications of such a technology can be imagined, all of them very important; however, for geological monitoring purposes, particularly interesting are the application for submarine, sea-bottom monitoring [3] and for borehole monitoring. Borehole temperature monitoring can be very important in volcanic and geothermal areas, both for sensing the heating of a volcanic system, which can highlight processes of magma migration in shallow reservoirs, and for geothermal exploration studies, in which it is very important to determine the thermal state at depth, and the heat flow through the geothermal gradient, in order to quantify the potential of the geothermal resource [4,5]. Temperature profiling in boreholes is usually determined using thermistor probes [6,7]. The majority of borehole temperature measurements are obtained as maximum-reading values acquired during logging runs or as continuously-recorded temperature surveys in wells. This method is generally used for wells drilled for many commercial purposes (exploration and production of hydrocarbons, minerals, and geothermal energy), as well as for water wells. Due to the conditions under which these data are obtained, and the purposes for which they are used, their accuracy is much lower than those obtained for measurements of the heat flow. Time constraints imposed by the commercial nature of these wells implies that wells may be logged during, or soon after, circulation of drilling fluids; during production of gas and fluids, at high logging speeds. Usually the maximum accuracy of commercial temperature logs is in the range of ±0.5 °C [8]. These data, obtained under dynamic conditions, must then be extrapolated to static conditions. [9,10,11] summarize the acquisition and uses of temperature surveys and temperature logging in the oil industry, and [6,12] in mineral exploration. Only recently it has been made possible to retrieve temperature surveys dedicated to geothermal exploration, monitoring and exploitation. Such surveys are mainly applied to low and medium enthalpy [13] with just few applications to deep wells devoted to high enthalpy or supercritical fluids studies, sometimes directly drilled into the magma ([14,15,16,17]; i.e., IDDP-Iceland, Unzen-Japan, CFDDP and DESCRAMBLE-Italy). Several authors [8,18,19,20], provided excellent reviews about the scientific measurement of heat flow, for both the technology and the methods of analysis. The logging technique is either “stop and go”, or the probe is lowered into the borehole at a controlled speed. Some experimental restrictions are associated with moving thermal probes, as follows [21]:

a) If not permanently installed and operating, simultaneous long-term and on-line temperature surveying for the entire length of the borehole is not feasible;
b) In the case of long, inclined (>30°) or horizontal boreholes, or boreholes that are directed upwards, conventional probing with moving temperature probes will fail for technical reasons;
c) Viscous mud has to be removed before thermal probe lowering, a procedure that entails considerable risk of damage to the entire borehole.

Continuous temperature-depth logs, especially when recorded in boreholes at thermal equilibrium conditions, provide detailed information on the subsurface thermal structure, which is very important for heat-flow measurements and rock thermal properties determination. When associated with independent thermal-conductivity determinations, thermal logging data may also allow to separate heat conduction from thermal convection effects. In addition, determining the main features of the thermal field in the shallow crust, is also valuable to characterize and quantify geothermal and hydrocarbon resources. Since 1981 researchers have tried to introduce and apply new temperature monitoring methods using optical fibers [21]. The Distributed Temperature Sensing (DTS) technique [22,23,24,25,26,27,28] is based on the Optical Time Domain Reflectometry (OTDR) concept [29,30,31,32]. This technique uses the optical fiber as the sensing element, with the intensity of the Raman back-scattered light of a laser pulse as a temperature dependent parameter. The method allows the instantaneous measurement of temperature along a fiber with an exact determination of the distance, fixed by the light velocity in the fiber. In terms of borehole logging, the DTS principle is completely different from conventional temperature logging systems, having a broad range of potential applications that cannot be easily obtained with conventional methods. For instance, it allows to monitor in the best way dynamic systems, because the temperature measurements are made simultaneously at all the depths in the borehole. The first application of the DTS technique in geoscience dates back 1992 [33]: an optical-fiber was lowered in a borehole and a temperature log was obtained under steady-state conditions. Another pioneering well-log application was realized in boreholes of the Grimsel Rock Laboratory of NAGRA (Wettingen, Switzerland), focused on measuring the impact of injected warm and cold fluids on the temperature profiles [34]. The main advantages of using fiber optics technologies for borehole monitoring are that the fiber has a low cost, and can be installed even in extremely corrosive and hot (up to 700 °C) environment settings; furthermore, the temperature can be measured repeatedly, almost instantaneously, and without physically disturbing the state of the borehole. These features make such technology particularly suitable for studying transient events. In general, DTS technology can be very useful for monitoring and interpretation of dynamic subsurface processes involving temperature changes [35].

A very important example are volcanoes, where deep processes involving magma, magmatic gases of hydrothermal fluids are generally accompanied by temperature changes at depth. In volcanic areas, accurate and continuous temperature monitoring at depth can be the key to discriminate the nature of unrest observations, and to forecast the possible evolution of volcanic processes, including eruptions.

In this paper, we will describe a DTS borehole sensing device, designed and realized to be installed into the pilot hole of the Campi Flegrei Deep Drilling Project (CFDDP), in the Bagnoli area (Naples West End). CFDDP [1,2] is an international research project aimed to understanding volcano dynamics at the Campi Flegrei caldera, as well as the mechanisms causing unrest phenomena associated to large uplift and subsidence (called bradyseism), often occurring in the last decades. One of the main goals of the project is also to install and progressively improve a reliable network of borehole observation systems, called ‘CFDO’ (Campi Flegrei Deep Observatory). The project core is essentially based on two wells: the first one, already drilled, reaches 500 m of depth (the pilot hole); the second, to be planned, will reach a depth of about 3500 m. The pilot hole was mainly aimed to study in detail the stratigraphy and eruptive history of the easternmost caldera border, whose sub-structure is the less known for lack of previous drillings (numerous, on the contrary, in the North and Western sectors, drilled in the period 1940–1985 for geothermal exploration purposes). This area is also the highest risk one, due to the extreme urbanisation and population density (it is located within the city of Naples). The Campi Flegrei Deep Drilling Project is sponsored and co-funded by the International Continental Drilling Program (see CFDDP website). The deep hole (about 3.5 km) is mainly aimed to study the thermal state, the rheology and the fluid-dynamics of the volcanic substructure, and to improve the knowledge about the earliest volcanism in the Neapolitan area. 

**Figure 1:**
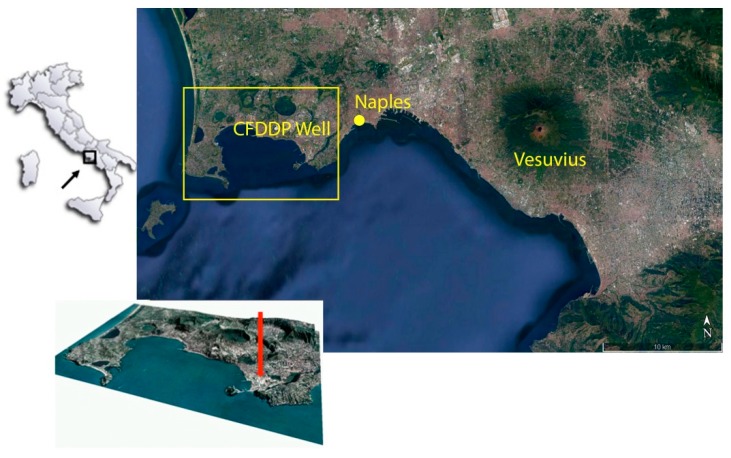
Map of the Neapolitan volcanic area in Southern Italy (Vesuvius, Campi Flegrei, Ischia island. The red square surrounds the Campi Flegrei caldera, containing part of large city of Naples. The yellow circle (and the red arrow in the small perspective view of the caldera) indicate the site of the Campi Flegrei Deep Drilling well.

De Natale et al. [36,37] analysed in detail the stratigraphy of the pilot hole, down to 500 m of depth. The pilot hole further hosted some specific experiments, aimed to determine the ‘in situ’ permeability and background tectonic stress [4]. Finally, the pilot hole and a subsidiary hole (200 m depths) have been drilled to host the first nucleus of the CFDO, which has been then improved by other borehole instruments (such as seismic arrays). A map of the area in which the pilot hole, completed at the end of December 2012, is located, can be seen in Figure 1.

We present here the borehole temperature sensing apparatus, belonging to the first nucleus of CFDO, as well as the results of a long-term monitoring campaign carried out along a 500 m-deep borehole located in Bagnoli, Campi Flegrei caldera, Italy, through a Raman scattering based DTS and a multi-mode optical fiber sensor cable capable of operation up to 150 °C.

## 2. Materials and Methods

### 2.1. The DTS Sensors

Raman scattering is a form of inelastic light scattering, originating from the interaction of light with molecular vibrations. In Raman-active molecules, the molecular vibration causes a fluctuation of the polarizability at the vibrational frequency, which is then periodically different from that of other parts of the medium, resulting in scattering [20]. In this process, energy is transferred between the incident photon and the vibration states of the molecule. In the most frequent case, the incident photon excites a molecular vibration and sheds energy in the process. As a result, the scattered photon carries a reduced amount of energy and its wavelength is increased in the process, which is referred to as Stokes Raman scattering. In the opposite case, vibrational energy is transferred to the scattered photon: such a process is known as anti-Stokes Raman scattering and the resulting scattered light appears at a shorter wavelength. Temperature sensors based on Raman scattering rely on the temperature sensitivity of the intensities of the anti-Stokes $I_{\mathrm{as}}$ and Stokes $I_{s}$ scatter. In particular Raman temperature systems commonly use the ratio $R\left(T\right)={\;I}_{\mathrm{as}}\left(T\right)/{\;I}_{s}\left(T\right)$, which is given by:

(1) $$R\left(T\right)=\left(\frac{k_{\mathrm{as}}}{k_{s}}\right){\left(\frac{\lambda_{s}}{\lambda_{\mathrm{as}}}\right)}^{4}\exp\left(-\frac{S_{\mathrm{LE}}}{T}\right)$$

In Equation (1), the coefficients $k_{\mathrm{as}\left(s\right)}$ depend on the Raman cross-section for the anti-Stokes (Stokes) bands, $\lambda_{\mathrm{as}\left(s\right)}$ are the wavelengths of the anti-Stokes and Stokes Raman scattered light, T is the absolute temperature, and $S_{\mathrm{LE}}$ defines the temperature sensitivity of the system, which is given by:

(2) $$S_{\mathrm{LE}}=h\cdot \nu_{R}\cdot c/k_{B}$$

**Figure 2:**
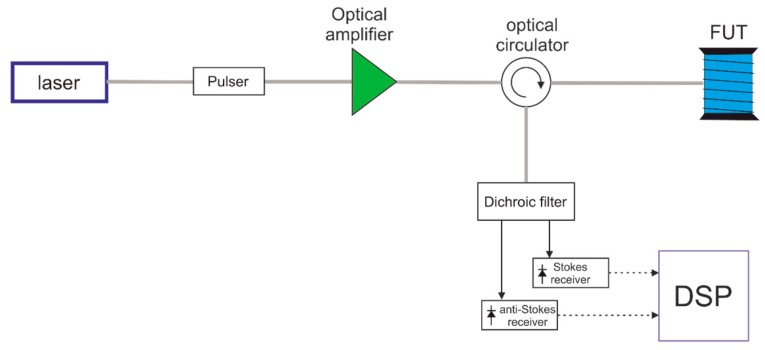
Schematic diagram of a Raman ratio distributed temperature sensor.

In Equation (2), h is the Plank’s constant, $\nu_{R}$ is the Raman shift, c is the speed of light in vacuum, and $k_{B}$ is the Boltzmann constant. For a typical $\nu_{R}=400{\;\mathrm{cm}}^{-1}$, $S_{\mathrm{LE}}$ amounts to 633.1 K [20]. Combining Equations (1) and (2), it comes out that the sensitivity of the Raman ratio R at around room temperature (T = 293 K) is about 0.74%/K for a typical silica-based glass. A schematic diagram of a Raman ratio distributed temperature sensors is shown in Figure 2.

In order to realize distributed temperature sensing in an optical fiber, an arrangement based on Optical Time-Domain Reflectometry (OTDR), such as the one shown in Figure 2, is typically employed. The light from a laser source is modulated by either an electro-optic or acousto-optic modulator, in order to provide probe pulses at wavelength λ_p. After optical amplification, these pulses are launched into the sensing fiber through an optical circulator. The backscatter light returned by the sensing fiber is separated into each of the Raman signal bands, trough of a dichroic filter [18]. The two Raman signals are sent onto separate detectors, while the resulting electrical signals are digitized and processed in order to convert the data into the form of temperature information.

**Figure 3:**
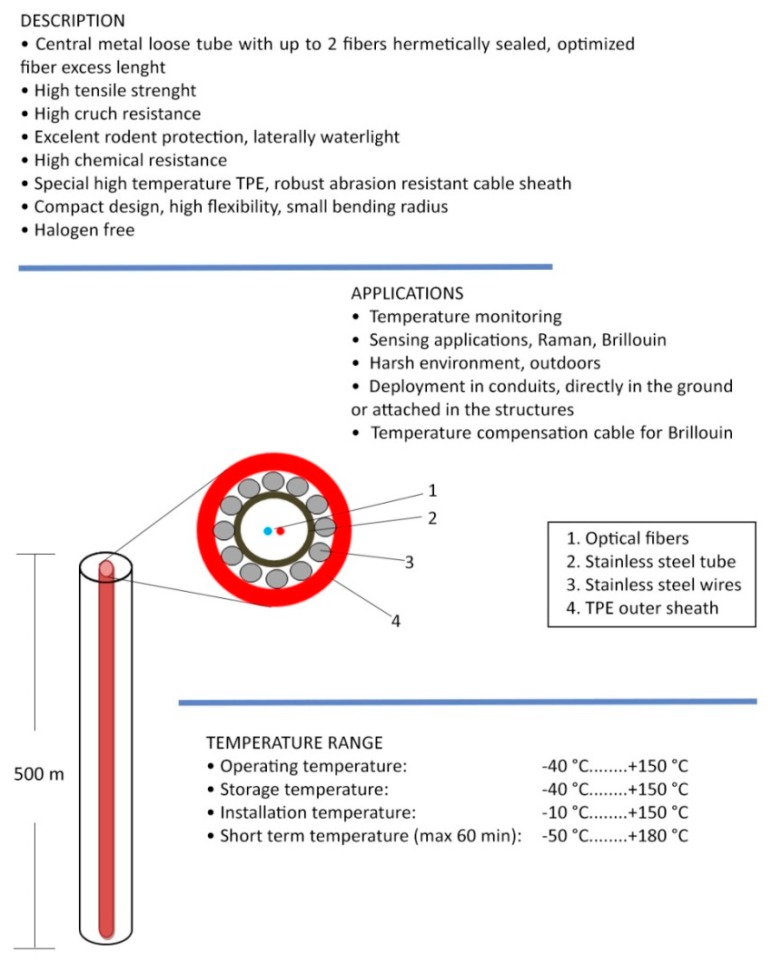
Small fiber optic mid temperature sensing cable, armored with stainless steel loose tube, stainless steel strength members and TPE outer sheath, fast thermal response up.

The DTS interrogation unit was assembled making use of a laser source at 785 nm. The minimum pulse width was 30 ns, resulting in a spatial resolution of about 3 m. The DSP provided a sampling resolution of 1 m and a number of accumulations up to $2^{23}$, while the acquisition time was (maximum) 4 min. A characterization of the DTS unit was carried out in order to determine the accuracy and repeatability of temperature measurements. The results of this activity indicate a temperature accuracy of ±1 °C for a fiber length of 2 km and $2^{21}$ accumulations. With the same fiber length and number of accumulations, the temperature resolution was 0.35 °C along the first 500 m, 0.5 °C up to 1 km, and 1 °C up to 2 km. Note that, the temperature accuracy was calculated as the maximum deviation between the actual temperature and the average temperature over a fiber length corresponding to the spatial resolution, while temperature resolution was calculated as the standard deviation of temperature over the same length. A schematic of the sensing cable is shown in Figure 3.

### 2.2. Well and Fiber Optic Design

**Figure 4:**
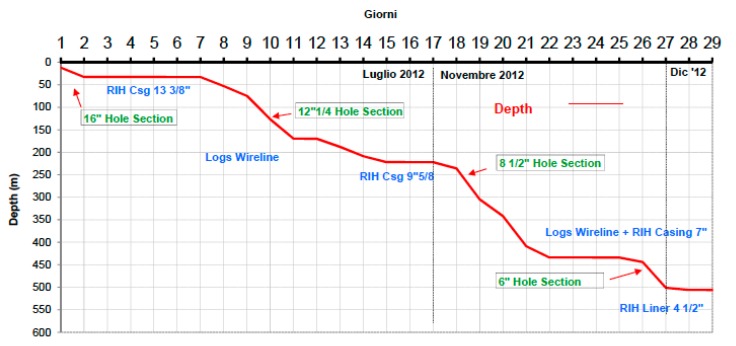
Well drilling diagram.

**Figure 5:**
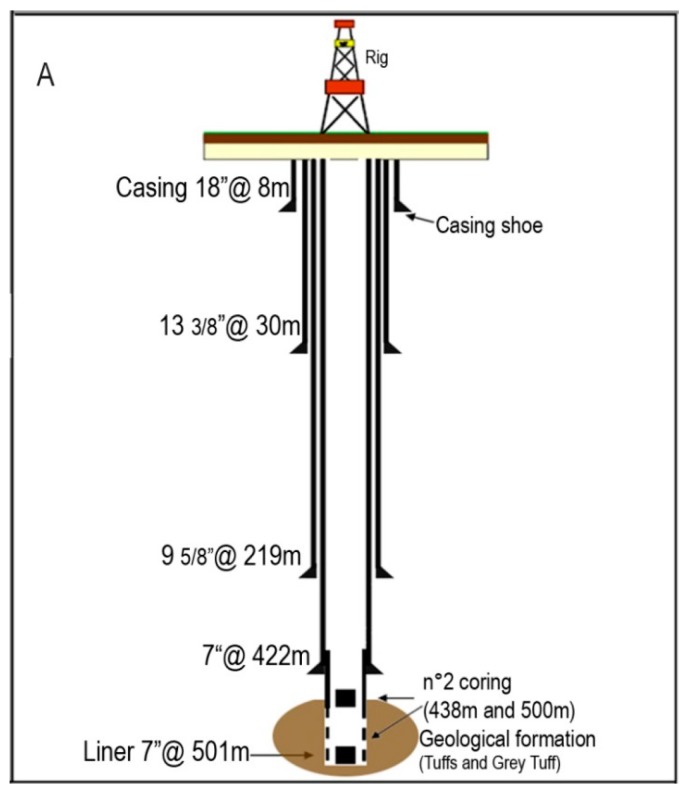

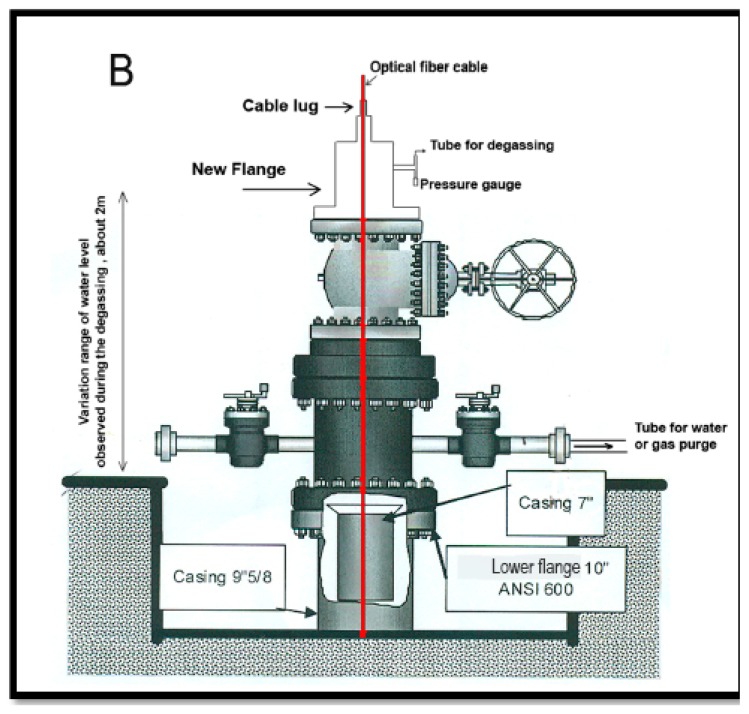

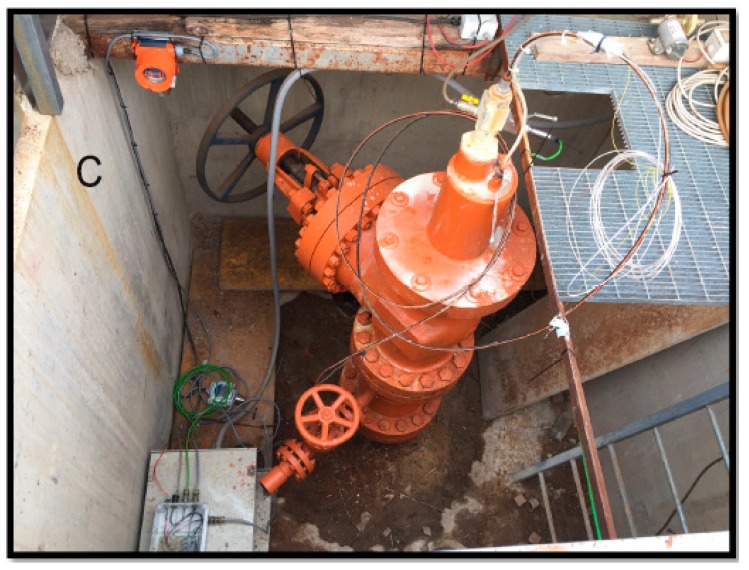
Schematic well design (**A**); well head scheme (**B**); photo of the well head with DTS inserted (**C**).

The Bagnoli1 Well, pilot hole of the Campi Flegrei Deep Drilling Project (CFDDP) has been drilled from July to December 2012. The well has been co-funded by INGV, ICDP (international Continental Drilling Program) and the EU-GEISER Project. Drilling has been realized in two parts, using two different Rigs. The first rig, used to drill 0–222 m (since July 15th to July 30th) was a Massenza MI60 equipped with blow out preventers. Then, the drilling was stopped until November 21st, when it started again with a new, more powerful rig: a Corsair 300 with a rotary table. The drilling reached 501 m of depth on December 2nd, then it was stopped there. Casing shoes were located at depths of 8, 30, 219 and 422 m of depth. A slotted liner completed the well down to the maximum depth, leaving an open hole section in the depth range 422–501. On December 3rd, a Leak Off Test was performed to measure the size of the minimum tectonic stress and the permeability, at the bottom open hole section [3,19]. Figure 4 shows the drilling diagram. Well after drilling (in April 2014) the fiber optics sensor has been inserted into the well. The installed cable, as shown in Figure 3, featured a stainless steel loose tube for optical fiber protection, stainless strength members and TPE outer sheet. The overall diameter of the installed cable was 3.8 mm, while its maximum tensile strength was 1500 N. The well design and the scheme and photo of the well head are shown in Figure 5.

## 3. Results and Discussion

One important aspect is the calibration of the sensing fiber. As previously described, Raman DTS extracts the temperature based on the intensity of the backscattered Stokes and Anti-Stokes signals. However, these two signals experience a different optical attenuation along the fiber. Since the difference between Stokes and Anti-Stokes optical loss (the so-called loss calibration parameter) is also depending on the optical fiber, it must be determined for each fiber individually in order to get accurate results. To this aim, the sensing cable shown in Figure 3 was put in a temperature chamber, and the loss calibration parameter was determined in order to eliminate any residual slope from the retrieved temperature.

Besides loss calibration, other parameters must be determined in order to retrieve the temperature profile with the greatest precision. In particular, indicating with $T_{0}\left(z\right)$ the temperature profile obtained after loss calibration, the final temperature profile is determined as:

(3) $$T\left(z\right)=A\cdot T_{0}\left(z\right)+B\cdot z+C$$

where *A* is the magnification, *B* is the slope and *C* is the offset. The three coefficients *A*, *B* and *C* were determined by keeping the sensing cable at a uniform temperature, except for a short length kept at a different (but known) temperature. In particular, the coefficient *B* was determined by applying a linear regression analysis to the temperature data along the unperturbed fiber, while the remaining coefficients *A* and *C* were determined by comparing the de-trended temperature with the known temperatures at the perturbed and unperturbed regions.

**Figure 6:**
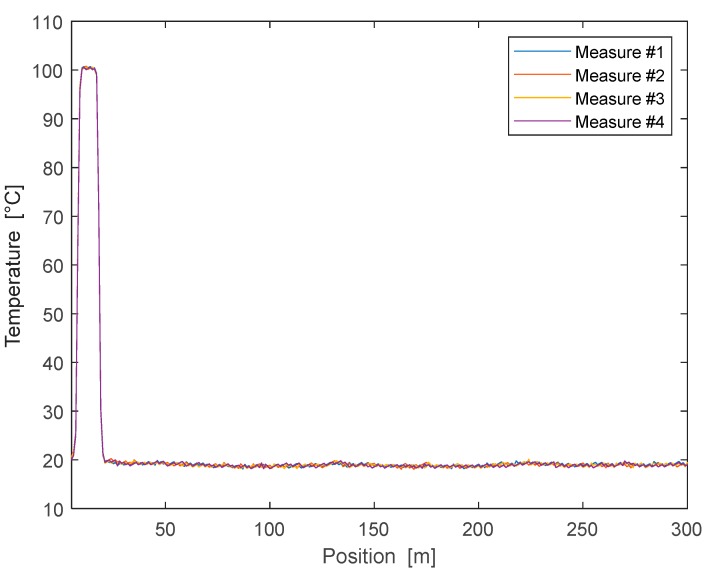
Temperature profile acquired using the assembled DTS in four successive runs.

As an example, we show in Figure 6 the temperature profile acquired in four successive runs, after performing the calibration procedure. For this test, a short section (20 m) of the fiber was immersed in boiling water while keeping the rest at room temperature (19.5 °C). From the figure, we can appreciate the good repeatability of the measurements, with a maximum deviation of the temperature equal to 1.07 °C along the uniform region, and 0.36 °C at the hot spot.

**Figure 7:**
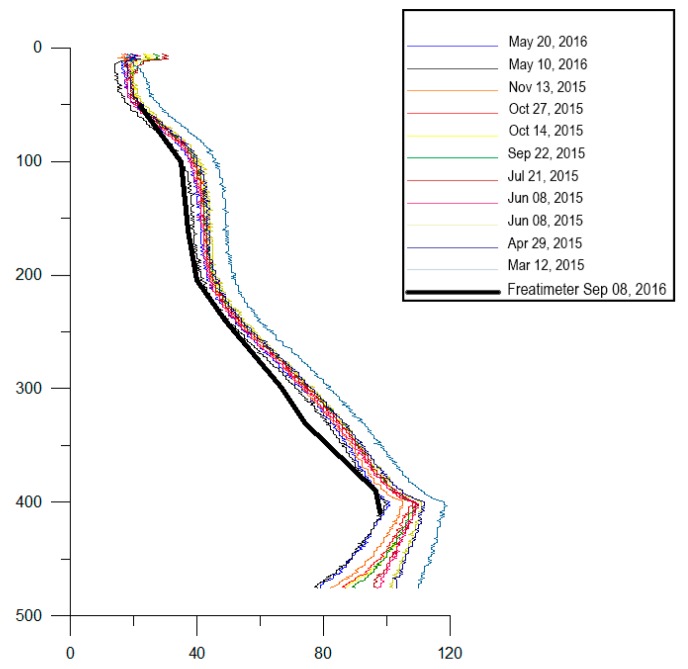
Temperature depth at different times along the CFDDP pilot hole.

After the DTS installation in the well, measurements have been obtained in several time periods (see Figure 7). In September 2016 a comparison has been made with the temperature values obtained using an electric freatimeter (thermo-couple). The temperature profiles shown here have been acquired during a period of about 15 months. Some of the temperature-depth plots obtained at different times are shown in Figure 7, as well as the plot obtained by the thermo-couple calibration test.

The very good agreement between the two temperature profiles, after more than one year of operation in a very harsh environment, is important and not obvious. In fact, tests of commercial fiber optics in very harsh environments are usually made in laboratory-controlled simulated conditions, and put in evidence degradation effects [37]. The main degrading factor in boreholes, known as ‘hydrogen darkening’, is mainly due to the hydrogen which binds to silica glass compound forming hydroxyls, causing increased attenuation of light passing in the fiber. In Table 1 we report the gas composition measured in the borehole before the fiber installation and at the end of the measurement period here shown (May–June 2016). For each measurement period, Table 1 reports the minimum and maximum gas concentration measured. As it is clear, the borehole environment is chemically aggressive, besides the high temperatures; it contains significant concentrations of H_2_S, and of hydrogen. Actually, the PTE outer sheath protection demonstrates effective to minimize the hydrogen darkening effect [38], and our test is then significant to demonstrate the feasibility of long term borehole monitoring with commercial fiber optics, provided they are appropriately protected with PTE sheaths.

The deployment of fiber-optics distributed temperature sensing then proved to be successful for temperature monitoring in boreholes. One of the main advantages of DTS technology is that continuous temperature profiles can be registered with high spatial and temporal resolution. This allows the accurate monitoring of dynamic subsurface processes involving temperature changes. Furthermore, the permanent installation of the sensor cable behind casing allows for full access to the well during the temperature measurement. Even abandoned and sealed wells can be monitored, which makes this method especially suitable for spatially dense, long-term thermal monitoring.

## 4. Conclusions

The optical sensing device here described represents the first reliable example of DTS installation for continuous monitoring of temperature all along a deep well, in a volcanic area. Changes of temperature at different depths are in fact a critical, although difficult to measure directly, datum for understanding the possible evolution of volcanic activity. Temperature changes at some depths have been often hypothesized during volcano unrest, by indirect methods; generally by changes of equilibria among different gas species [39,40]. Such changes can reflect either the intrusion of magma at shallow depth or simply the rising of hot gases from deeper levels into shallow aquifers. Discriminating the source of unrest at volcanic areas, mainly at calderas where large geothermal systems generally exist, is one of the main problems for short term volcanic hazard assessment and eruption forecast. Actually, this problem is afforded by the use of seismological, geochemical and ground deformation data. Temperature change measurements along deep well profiles, in a continuous way and with high sensitivity, can add a new, powerful data set able to strongly constrain such a fundamental question in volcanic areas. At Campi Flegrei, the measurements shown here demonstrate that, in the last years, there have not been considerable temperature changes in the Bagnoli zone on the easternmost border of the caldera, except perhaps a modest decrease of temperature occurring at all depths along the well profile. This result was not obvious, because several recent models hypothesized a progressive increase of temperature, of several tens degrees (°C), in the caldera substructure ([19] and references therein). Such an increase of temperature at depth, which had been indirectly inferred from geochemical analyses of fluids in the Solfatara-Pisciarelli fumaroles, is thus, if reliable, likely to be confined in that sector of the caldera. This would be in fact the only possibility to hypothesize a progressive increase of temperature in the fumarole areas, since few kilometers farther, very close to the easternmost border of the caldera, the opto-electronic temperature sensor installed in the CFDDP pilot hole demonstrates, on the contrary, that temperature decreases at all depths within the first 500 m. Regarding the stability and reliability of our system, it can be proven by the observation that the temperature profile measured by the DTS, after more than one year from the installation and first measurements, still coincides, to within the expected uncertainty, with the values obtained by the thermocouple probe inserted into the well. This also demonstrates the effectiveness of PTE sheath outer protection, we used in our cable. In the present configuration, the DTS measurements will be very important to detect any possible temperature change at depth, reflecting for instance the rise of magma or magmatic fluids from deeper levels to shallower ones. The opto-electronic sensor here designed and installed, in a very harsh environment with temperature up to 120 °C and very aggressive fluids, also demonstrates that fiber optics are the best option for sensors to be installed in deep wells, in critical environmental conditions and high temperature. It is in fact possible, using fiber optics highly temperature resistant, to build sensors resisting to temperatures as high as 800 °C. Such extreme temperatures are very close to the magma temperature and much higher than the supercritical temperature, which can hold in the ductile part of the volcanic substructure, tipically around 400–500 °C.

Actually, a number of new technologies are going to be developed for geothermal exploration and logging in supercritical regimes [41,42]. Supercritical conditions, involving very high temperature and pressure, as well as very aggressive environmental conditions, are recently considerated as the ‘new frontier’ for the future geothermal plants in active volcanoes, capable of an order of magnitude step in the electrical generation capacity. For these reasons, besides significantly improving the knowledge and monitoring of volcanic structures [2], a number of projects, aimed to geothermal exploitation of supercritical fluids, have been planned or are going on [43]. The pioneering project aimed to exploit supercritical conditions has been the Icelandic Deep Drilling Project (IDDP) [44]. Fiber optics measurement tools, and in particular DTS technology, appear to be the most suitable and performing ones to work in such hot and aggressive environments.

In addition to temperature measurements and logging, deep borehole sensors based on DTS or Distributed Strain Sensors (DSS), can be a formidable tool to record microseismicity directly at the seismogenic (or near-seismogenic) depths, in a deep environment well isolated from the surface noise. Such a technology, or else the Bragg’s gratings one, used to build opto-electronic seismometers (i.e., [1,44]) in form of distributed or dense depth seismometer arrays, can represent a formidable tool for a step forward in the monitoring, study and understanding of volcanic, tectonic and anthropic induced microseismicity. It can be also very useful, during oil and gas exploration and extraction, for accurate monitoring of the induced microseismity and for increasing the precision in seismic tomography at depth. Such technologies thus open very innovative perspectives in volcano monitoring, where seismicity and temperature are key parameters to interpret what is going on; and to detect, in particular, the rising of deep fluids, either gas exhaled from magma or magma itself.

## Figures and Tables

**Table 1:** Main gas concentrations measured in the CFDDP pilot hole before the fiber installation and after the period of DTS measurements shown in the paper.

Well	Date		CO_2_	H_2_S	^36^Ar	^40^Ar	O_2_	N_2_	CH_4_	H_2_	He	CO
CFDDP	Oct/Nov 2014	max	992577	16.12	0.00965	3.38	23.96	6625	10402	12118	7.52	36.6
CFDDP		min	971432	5.74	0.00638	2.88	5.13	2059	3253	2078	2.23	0.00
CFDDP	Jul/Nov 2016	max	977439	44.40	n.a.	119.39	78.79	5209	8448	10102	13.97	n.a.
CFDDP		min	954402	28.77	n.a.	22.85	14.83	3042	5533	2671	7.67	n.a.
